# Direct and rapid measurement of hydrogen peroxide in human blood using a microfluidic device

**DOI:** 10.1038/s41598-021-82623-4

**Published:** 2021-02-03

**Authors:** R. Gaikwad, P. R. Thangaraj, A. K. Sen

**Affiliations:** 1 Micro Nano Bio-Fluidics Unit, Fluid Systems Laboratory, Department of Mechanical Engineering, Indian Institute of Technology Madras, Chennai, 600036 India; 2 Department of Cardiothoracic Surgery, Apollo Hospital, Chennai, 600006 India

**Keywords:** Biomedical engineering, Chemical engineering

## Abstract

The levels of hydrogen peroxide (${\mathrm{H}}_{2}{\mathrm{O}}_{2}$) in human blood is of great relevance as it has emerged as an important signalling molecule in a variety of disease states. Fast and reliable measurement of ${\mathrm{H}}_{2}{\mathrm{O}}_{2}$ levels in the blood, however, continues to remain a challenge. Herein we report an automated method employing a microfluidic device for direct and rapid measurement of ${\mathrm{H}}_{2}{\mathrm{O}}_{2}$ in human blood based on laser-induced fluorescence measurement. Our study delineates the critical factors that affect measurement accuracy—we found blood cells and soluble proteins significantly alter the native ${\mathrm{H}}_{2}{\mathrm{O}}_{2}$ levels in the time interval between sample withdrawal and detection. We show that separation of blood cells and subsequent dilution of the plasma with a buffer at a ratio of 1:6 inhibits the above effect, leading to reliable measurements. We demonstrate rapid measurement of ${\mathrm{H}}_{2}{\mathrm{O}}_{2}$ in plasma in the concentration range of 0–49 µM, offering a limit of detection of 0.05 µM, a sensitivity of 0.60 µM^−1^, and detection time of 15 min; the device is amenable to the real-time measurement of ${\mathrm{H}}_{2}{\mathrm{O}}_{2}$ in the patient’s blood. Using the linear correlation obtained with known quantities of ${\mathrm{H}}_{2}{\mathrm{O}}_{2}$, the endogenous ${\mathrm{H}}_{2}{\mathrm{O}}_{2}$ concentration in the blood of healthy individuals is found to be in the range of 0.8–6 µM. The availability of this device at the point of care will have relevance in understanding the role of ${\mathrm{H}}_{2}{\mathrm{O}}_{2}$ in health and disease.

## Introduction

Reactive oxygen species (ROS), such as superoxide anion (O_2_^−^), hydrogen peroxide (H_2_O_2_), and hydroxyl radical (HO·), are free radical and reactive molecules derived from the partial reduction of molecular oxygen^1^. Cellular ROS are produced endogenously due to mitochondrial oxidative phosphorylation, or from interactions with exogenous sources such as xenobiotic compounds. Reactive oxygen species (ROS) play an essential role in regulating several signalling pathways through interaction with critical signalling molecules^2^. ROS appears as a key element in a broad range of physiological and pathophysiological processes^3^ such as proliferation^4,5^, metabolism, differentiation, and survival, antioxidant and anti-inflammatory response, iron homeostasis, and DNA damage response^5^. When ROS overpowers the antioxidant defence system either through an increase in ROS levels or a decrease in the cellular antioxidant capacity, oxidative stress occurs^6^. Oxidative stress results in damage to nucleic acid, proteins, and lipids^7^ and can contribute to carcinogenesis^8^, neurodegeneration^9^, atherosclerosis, diabetes^6^, and aging^10^.

Hydrogen peroxide (${\mathrm{H}}_{2}{\mathrm{O}}_{2}$) is one of the important ROS which is produced due to the incomplete reduction of oxygen in the metabolism process^11^ and most cells in the human body generate ${\mathrm{H}}_{2}{\mathrm{O}}_{2}$ from superoxide^1,12^. ${\mathrm{H}}_{2}{\mathrm{O}}_{2}$ is uncharged and stable in aqueous solution and its uncharged nature helps it to diffuse and transport across the cell membrane, enabling cellular signalling away from the site of production. Moreover, ${\mathrm{H}}_{2}{\mathrm{O}}_{2}$ has a longer lifetime in comparison to other free radicals, which allows a diffusion distance up to a few milimeters^13^. ${\mathrm{H}}_{2}{\mathrm{O}}_{2}$ which is diffused out of a cell triggers cell migration, immunity generation, and cellular communication. Literature shows the usage of ${\mathrm{H}}_{2}{\mathrm{O}}_{2}$ as an intercellular and intracellular signalling molecule. For example, activated phagocytes at the site of inflammation generate ${\mathrm{H}}_{2}{\mathrm{O}}_{2}$ to control cell proliferation and platelet aggregation^7,8^. However, an imbalance in the level of ${\mathrm{H}}_{2}{\mathrm{O}}_{2}$ to the antioxidants can have a detrimental effect leading to damage of nucleic acids^13^ and diseases related to oxidative stress^14^. Recently, the role of ${\mathrm{H}}_{2}{\mathrm{O}}_{2}$ in the regulation of gasotransmitters, such as nitric oxide, carbon monoxide, and hydrogen sulphide is explained^3^. Therefore, measurement of ${\mathrm{H}}_{2}{\mathrm{O}}_{2}$ level in the blood has importance in understanding the role of ${\mathrm{H}}_{2}{\mathrm{O}}_{2}$ as a potential biomarker in health and disease. Moreover, in addition to its profound significance in biological studies,${\mathrm{H}}_{2}{\mathrm{O}}_{2}$ has relevance in other applications such as food and paper industries, environmental analysis, mineral processing, and fuel cells^15^.

Blood cells including red blood cells produce ${\mathrm{H}}_{2}{\mathrm{O}}_{2}$ from multiple sources but the level of intracellular ${\mathrm{H}}_{2}{\mathrm{O}}_{2}$ is maintained as 10 nM or less due to the catalase and peroxidases^1^. The plasma ${\mathrm{H}}_{2}{\mathrm{O}}_{2}$ is mainly contributed by NOXs (nicotinamide adenine dinucleotide phosphate oxidase) on the surface of phagocytes and endothelial cells and xanthine oxidase bound to endothelial cells with a small contribution from autoxidation of small molecules. ${\mathrm{H}}_{2}{\mathrm{O}}_{2}$ can leave and enter the cells through aquaporins and the prevailing direction of ${\mathrm{H}}_{2}{\mathrm{O}}_{2}$ movement into the cells. Measurement of the concentration of ${\mathrm{H}}_{2}{\mathrm{O}}_{2}$ in the human blood has been attempted but it remains controversial since there is a large variation in the results^1^. The concentration of ${\mathrm{H}}_{2}{\mathrm{O}}_{2}$ in the human blood will depend on the dynamics of its production and its removal. It appears that the claims of steady-state plasma ${\mathrm{H}}_{2}{\mathrm{O}}_{2}$ concentration in the mid mM to high mM range was overestimated, caused by interfering factors and inadequate method and instrumentation^1^. Various compounds and contaminants present in blood plasma can react with the assaying dye leading to such overestimated values. Similarly, very low values of plasma ${\mathrm{H}}_{2}{\mathrm{O}}_{2}$ concentration ≤ 0.25 mM was also reported which could be due to the limitations of the measurement techniques^1^. The plasma ${\mathrm{H}}_{2}{\mathrm{O}}_{2}$ concentration in healthy humans appears to fall into two groups: the first group reports that the measured concentration < 10 µM and in the second group, the measured concentration is between 20 and 40µM^1^. The levels indicated by the second group is not expected for healthy individuals as ${\mathrm{H}}_{2}{\mathrm{O}}_{2}$ concentration in that range is stressful to cells, although not completely toxic. From an analysis of literature and kinetics, the most probable range for plasma ${\mathrm{H}}_{2}{\mathrm{O}}_{2}$ is 1–5 µM^1,17–20^.

Various techniques for the detection of ${\mathrm{H}}_{2}{\mathrm{O}}_{2}$ have been reported, such as titration^16^, spectroscopy^17^, fluorescence^18^, chemiluminescence^19^, spectrophotometry^20^, and electrochemical^21^ methods. The conventional methods such as titrimetry, chemiluminescence, and spectrophotometry are not suitable because of several drawbacks such as low sensitivity, selectivity, long detection time, and complicated instrumentation involved^22^. These methods involve the manual handling of the sample, which is time-consuming and can lead to the degradation and/or change in the levels of the native hydrogen peroxide, therefore affecting the reliability of measurements. The electrochemical methods that are based on the electron transfer are preferred over the conventional methods owing to the ease of operation and integration. However, such methods have not been applied so far to the human blood sample and inherent disadvantages such as high overpotential^23^ and slow electron transfer^24^ leading to low sensitivity, poor selectivity, and metal fouling. Electrochemical methods based on the horseradish peroxidase (HRP) enzyme for catalytic decomposition of peroxide are more sensitive and are widely preferred^22,24^. A nanochannel platform involving HRP enzyme and carbodiimide coupling chemistry for the detection of ${\mathrm{H}}_{2}{\mathrm{O}}_{2}$ was reported^23^. In optical ${\mathrm{H}}_{2}{\mathrm{O}}_{2}$ detection, most systems involve the use of optical indicator probes. In the HRP based fluorometric assay, a colourless and non-fluorescent compound is oxidised to a fluorescent substance by hydrogen peroxide in the presence of HRP. The latter method is simple and sensitive because of the formation of a strong fluorescent product and its wide usage in a range of systems and condition^25,26^. But such methods have not been applied to human blood sample, involve manual handling and are not amenable to in-situ and continuous monitoring, which is one of the goals of the present work.

Despite the above developments in the sensor technology for the detection of ${\mathrm{H}}_{2}{\mathrm{O}}_{2}$, it is found that the concentration of measured ${\mathrm{H}}_{2}{\mathrm{O}}_{2}$ in blood spans several orders of magnitude from µM to mid mM, which can be attributed to the following: (a) there is a lack of understanding of how the concentration of ${\mathrm{H}}_{2}{\mathrm{O}}_{2}$ dynamically change between the sample collection and measurement time points, (b) most of the methods and instrumentation currently being used are inadequate to accurately measure ${\mathrm{H}}_{2}{\mathrm{O}}_{2}$ levels in the blood. Further, most of the methods reported in the literature so far are not amenable to direct and rapid measurements leading to inconsistent and delayed results. Microfluidics is a proven technology for rapid and automated measurement of a range of biomolecules^26,27^. In the present work, we address the above issues by identifying some of the critical factors that affect dynamic change in ${\mathrm{H}}_{2}{\mathrm{O}}_{2}$ in the blood plasma and develop a method based on an integrated microfluidic device for accurate and rapid measurement of exogenous ${\mathrm{H}}_{2}{\mathrm{O}}_{2}$ in blood plasma. We employ on-chip blood plasma separation to obtain cell-free plasma and dilute the plasma with a buffer to reduce the interference of blood cells and plasma proteins with ${\mathrm{H}}_{2}{\mathrm{O}}_{2}$.The device is used for measurement of the actual quantity of exogenous ${\mathrm{H}}_{2}{\mathrm{O}}_{2}$ in blood plasma to develop the correlation between FL intensity and ${\mathrm{H}}_{2}{\mathrm{O}}_{2}$ concentration. The developed correlation is then used to measure the concentration of endogenous ${\mathrm{H}}_{2}{\mathrm{O}}_{2}$ concentration in blood of healthy individuals.

## Experimental

### Device concept and design

**Figure 1:**
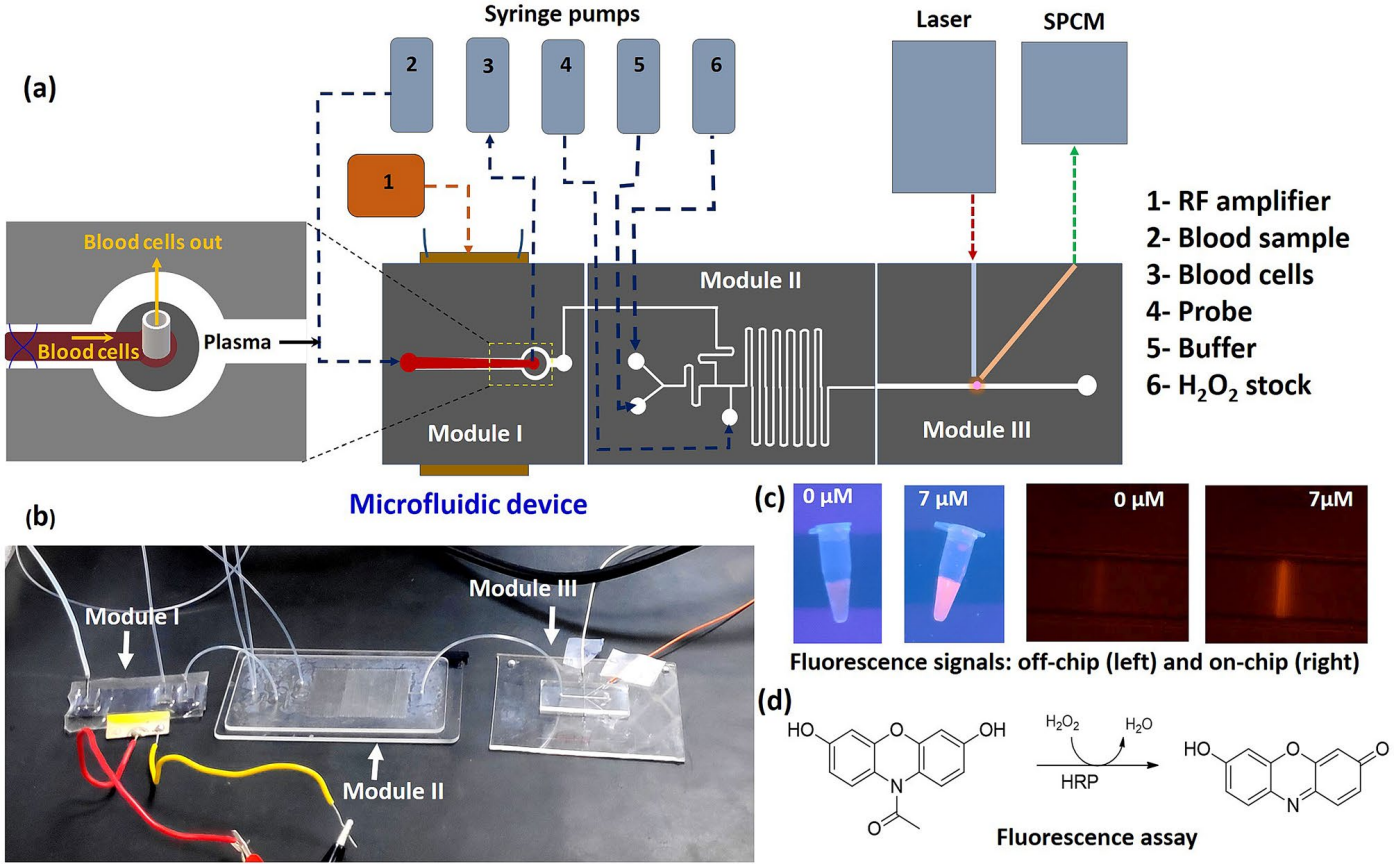
(**a**) Schematic of the integrated microfluidic device with the different modules (module I—blood plasma separation, module II—mixing and incubation, and module III—optical detection) and experimental setup, (**b**) photograph of the actual device showing the different modules, (**c**) Representative images showing FL signals from H_2_O_2_ + probe mixture in Eppendorf tube and microchannel and, (**d**) Probe chemistry.

The integrated microfluidic device for the detection of hydrogen peroxide (${\mathrm{H}}_{2}{\mathrm{O}}_{2}$) in the human blood consists of three different modules (see Figs. 1a,b, S2a). In Module I, cell-free plasma is obtained by separating the plasma and blood cells from whole blood using acoustophoresis. The separated plasma from module I is then infused into module II which serves the purpose of mixing and reaction, where reagents blood plasma, exogenous ${\mathrm{H}}_{2}{\mathrm{O}}_{2}$, Amplex Red substrate and horseradish peroxidase (HRP) are mixed and incubated. In the presence of ${\mathrm{H}}_{2}{\mathrm{O}}_{2}$, the HRP oxidises the Amplex Red substrate to form resorufin which is a fluorescent compound, which is transported to module III and detected using suitable optics.

The design of the acoustics-based blood plasma separation module (Figs. 1a and S2a) is reported elsewhere^28^. The width of the main channel is 300 µm to support half-wave at a frequency of 1.91 MHz, as calculated from theory and confirmed in experiments. In the present study, the flow rate of the whole blood sample is kept constant at 20 µL min^−1^ and acoustic energy density is 14.9 J m^−3^ to obtain cell-free plasma at a flow rate of 1.0 µL min^−1^. The function of the acoustic device is briefly described in Section S1 of “Supplementary Material”. The serpentine channel for the mixing and incubation module (Figs. 1a and S2a) is designed based on diffusion coefficients of the fluids involved, flow rates, and the incubation time as follows. The experiments are performed with the assay buffer and human blood plasma and in both cases, the exogenous concentration of $${\mathrm{H}}_{2}{\mathrm{O}}_{2}$$ is varied by adding the buffer to $${\mathrm{H}}_{2}{\mathrm{O}}_{2}$$ stock solution of 10 µM concentration, via the mixing channel. The diffusion coefficient of liquid $${\mathrm{H}}_{2}{\mathrm{O}}_{2}$$ in the assay buffer is taken as^29^ *D*_*s*_ = 1.4 × 10^−5^ cm^2^ s^−1^. The serpentine channel has a square cross-section of dimension 100 × 100 µm, which gives a diffusion time (τ $$={w}^{2}/{D}_{s})$$ of 7.14 s, where $$w$$ is the width of the channel. In experiments with buffer, the total flow rate of $${\mathrm{H}}_{2}{\mathrm{O}}_{2}$$ stock and buffer is kept constant at 3.8 μL min^−1^ while the individual flow rates are varied to obtain the different final concentrations of $${\mathrm{H}}_{2}{\mathrm{O}}_{2}$$, as shown in Table S1. In experiments with centrifuged plasma, the total flow rate of $${\mathrm{H}}_{2}{\mathrm{O}}_{2}$$ stock, buffer, and undiluted plasma is kept constant at 3.8 μL min^−1^. while the flow rate of the undiluted plasma is kept fixed at 0.54 μL min^−1^ (1:6 dilution of plasma is obtained on-chip by infusing additional buffer along with $${\mathrm{H}}_{2}{\mathrm{O}}_{2}$$ stock), the individual flow rates of the buffer and $${\mathrm{H}}_{2}{\mathrm{O}}_{2}$$ stock is varied (with a total flow rate of 3.26 μL min^−1^) to achieve the different final concentrations of $${\mathrm{H}}_{2}{\mathrm{O}}_{2}$$ (see details in Section S2 and Table S1 in “Supplementary Material”). In experiments with on-chip separated plasma in the integrated device, the plasma flow rate is maintained at 1.0 μL min^−1^, and the sum of the individual flow rates of the buffer and $${\mathrm{H}}_{2}{\mathrm{O}}_{2}$$ stock is varied, with a total flow rate of 2.8 μL min^−1^, to achieve the different final concentrations of $${\mathrm{H}}_{2}{\mathrm{O}}_{2}$$, as shown in Table S1. The velocity ($$u$$) of the stock and buffer mixture in the channel is 6.33 mm s^−1^ and therefore the corresponding diffusion length ($$L= u\tau $$) is 45 mm suggesting a mixing channel of length $$\ge $$ 45 mm. Next, the probe (HRP + Amplex Red substrate) is infused into the channel at a flow rate of 0.2 μL min^−1^, giving a total probe + $${\mathrm{H}}_{2}{\mathrm{O}}_{2}$$ flow rate of 4 μL min^−1^. Since the major constituent of the probe is the assay buffer, the probe and $${\mathrm{H}}_{2}{\mathrm{O}}_{2}$$ mix quickly during incubation. The length required for mixing is taken as the length required for the incubation, to ensure the probe and $${\mathrm{H}}_{2}{\mathrm{O}}_{2}$$ reaction to complete. Since the $${\mathrm{H}}_{2}{\mathrm{O}}_{2}$$ assay requires a minimum incubation time of 15 min for the reaction to complete, as found later in the “Results and discussion” section, a minimum length of the serpentine channel is maintained as 6.0 m to ensure adequate mixing and incubation.

The width and depth of the fluidic channel in the optical detection module (Fig. 1a and S2a) are 100 µm and 150 µm, respectively. Two different optical fibre grooves, of width and depth 150 µm are used to incorporate the excitation fibre (10/125 µm) and FL collection fibre (62.5/125 µm). The excitation fibre groove is placed perpendicular to the flow channel and the FL collection fibre groove is placed at an angle of 45° to the excitation fibre groove to minimise the direct exposure of the detector to the excitation light coming from the laser source. A minimum gap of 50 µm is maintained between the edges of the optical grooves and the flow channel sidewalls to minimize the attenuation of the excitation and emission signals. An index matching liquid, of refractive index $(\mathrm{RI})=1.458$, matching with the RI of fiber-glass, is filled between the edge of the fibres and the edges of the fibre grooves to prevent scattering of signals. At a total flow rate of 4 μL min^−1^ through the detection channel, the sample crosses the laser beam at a velocity of 4.4 mm s^−1^. For a laser beam of width 40 µm in the channel, the sample residence time is 8.18 ms, which allows the signal to be easily captured by a high-speed detector.

### Device fabrication and setup

Module I (blood plasma separation module) of the device is fabricated in silicon and glass substrates. Following photolithography, the channels (of 200 µm depth) are etched on a 500 µm thick silicon wafer (Semiconductor Technology and Applications, USA) using deep reactive ion etching (DRIE) technique. A borosilicate glass slide (Semiconductor Technology and Applications, USA) of 500 µm thickness is then bonded with the DRIE-etched silicon wafer by anodic bonding to seal the channels. A detailed procedure followed for the fabrication of the blood plasma separation module is reported elsewhere^28^. Module II (mixing and incubation module) and Module III (optical detection module) are machined in Polymethyl methacrylate (PMMA) using a CNC micro-milling machine (Minitech machinery, USA) as per the design outlined in the previous section. The required channel length of 6.0 m is accommodated on a PMMA sheet of size 7.5 cm × 3.5 cm. The PMMA layer with machined channels is bonded with a planar PMMA layer by first exposing both the surfaces to chloroform (≥ 99% stabilised, Ranken Chemical, India) for 2 min, by maintaining a 2 mm gap between the chloroform interface and PMMA surface, and then applying a pressure of 1.5 tons while exposing to a temperature of 65 °C for 30 min using a thermal–hydraulic press (Specac Ltd, UK). The roughness and optical transmittance of PMMA channels after machining, chloroform exposure, and heat treatment are discussed in the Supplementary Material Section S3.

A schematic and photograph of the experimental setup are shown in Fig. 1a. In on-chip blood plasma separation, the whole blood sample is infused into the device and concentrated blood cells are extracted out of the device using high-performance syringe pumps (Cetoni GmbH, Germany). For the acoustic actuation, the RF signal is generated using a function generator (SMB100A, Rohde & Schwarz, Germany), amplified using an amplifier (75A100A, Amplifier Research, USA) and the amplified signal is supplied to a lead-zirconate-titanate (PZT) transducer (Sparkler Ceramics, India) attached to the bottom of the silicon substrate using epoxy glue. The operating frequency is 1.91 MHz, and the power input is 206 mW. Depending on the experiments, different samples and reagents—buffer, ${\mathrm{H}}_{2}{\mathrm{O}}_{2}$ stock, plasma, and probe are infused into the device using syringe pumps (Cetoni GmbH, Germany) at the respective flow rates (Table S1). The fluidic connection between the pumps and the device inlets/outlet ports and the drain reservoir and between the different modules is established using polyethylene tubing (see Fig. 1b). A laser source (Wave Form Systems, Inc., USA) of 532 nm wavelength and 5 mW power is used as the excitation source and the fluorescence (FL) signal is detected using a single-photon counting module (SPCM) detector (50A/M, Thorlabs Inc., USA). A standard single-mode fibre (10/125 µm) carries the laser beam from the source to the channel and multimode fibre (62.5/125 µm) is used to carry the FL signal to a highly sensitive single-photon counting module (SPCM). The compound resorufin interacts with the laser beam and generates an FL signal with a peak at 590 nm. A bandpass filter (ET575/50m, Chroma Technology Corp. USA) is used to eliminate the background signal. The system involves minimal human intervention for operation and is suitable for real-time measurements.

## Results and discussion

### Demonstration of the assay: measurement of ${\mathbf{H}}_{2}{\mathbf{O}}_{2}$ in buffer off-chip and on-chip

As discussed in the “Materials and methods” section, a mixture of amplex red and horseradish peroxidase (HRP) is used as the chemical probe for the detection of ${\mathrm{H}}_{2}{\mathrm{O}}_{2}$ in buffer and plasma. In the presence of amplex red, HRP reacts with ${\mathrm{H}}_{2}{\mathrm{O}}_{2}$ to produce a fluorescent (FL) compound—resorufin (Fig. 1d). The FL compound is detected by exciting it with a laser and detecting the emission signal using a photodetector, as described earlier. The representative images of fluorescence signal in the Eppendorf tube and inside a microfluidic channel are shown in Fig. S2b and Fig. 1c (see details in the Supplementary Material Section S4).

First, we perform experiments to demonstrate the FL assay and determine the minimum incubation time required, by measuring ${\mathrm{H}}_{2}{\mathrm{O}}_{2}$ in buffer using a 96-well plate. ${\mathrm{H}}_{2}{\mathrm{O}}_{2}$ stock is mixed with the buffer to achieve ${\mathrm{H}}_{2}{\mathrm{O}}_{2}$ concentration $(c)$ in the range of 0–7 µM. The buffer containing ${\mathrm{H}}_{2}{\mathrm{O}}_{2}$ is mixed with the probe and the mixture is incubated for 15 min and FL intensity is measured using a plate reader (see Fig. S3). The relative volumes of the buffer and ${\mathrm{H}}_{2}{\mathrm{O}}_{2}$ stock solution used to achieve different concentrations of ${\mathrm{H}}_{2}{\mathrm{O}}_{2}$ is given in Table S1. Inset in Fig. S3 shows that the peak FL intensity is observed at an emission wavelength of 590 nm. The normalized FL intensity versus ${\mathrm{H}}_{2}{\mathrm{O}}_{2}$ concentration (see Fig. S3) is correlated as, ${I}^{*}=1.04 c$ (with ${R}^{2}=0.92$). Here a general definition of the normalized intensity ${I}^{*}=\left(\mathrm{I}-{\mathrm{I}}_{\mathrm{o}}\right)/{\mathrm{I}}_{\mathrm{en}}$, where $\mathrm{I}$ is the FL intensity measured at a particular concentration of ${\mathrm{H}}_{2}{\mathrm{O}}_{2}$, and ${\mathrm{I}}_{\mathrm{o}}$ is the FL intensity of the probe in buffer and ${\mathrm{I}}_{\mathrm{en}}$ is the FL intensity of the endogenous ${\mathrm{H}}_{2}{\mathrm{O}}_{2}$, i.e. with only plasma and probe but without any externally added ${\mathrm{H}}_{2}{\mathrm{O}}_{2}$). For buffer experiments, ${\mathrm{I}}_{\mathrm{en}}$ is the same as that of ${I}_{o}$ as there is no endogenous ${\mathrm{H}}_{2}{\mathrm{O}}_{2}$ present. Inset in Fig. S3 shows that a minimum of 15 min of incubation time is required for the completion of the assay reaction since we did not observe any significant change in the FL intensity when the buffer + probe mixture is incubated for more than 15 min (see inset in Fig. S3).

Although FL based detection is possible using a 96-well plate reader, it involves manual handling of the sample, and exposure to the light and ambient and hence will lead to a faster and much higher degradation in the ${\mathrm{H}}_{2}{\mathrm{O}}_{2}$ level in the sample. On the other hand, the system presented here is a closed system, and it needs human intervention only while infusing the blood sample, which minimizes the processing time and exposure to the external environment. Therefore our system allows monitoring the ${\mathrm{H}}_{2}{\mathrm{O}}_{2}$ in real-time which is not possible using the conventional 96-well plate configuration. Further, the use of a 96-well plate would involve a manual centrifugation step to remove the blood cells for preventing interference with the optical reader whereas in the present system the plasma separation is achieved on-chip.

**Figure 2:**
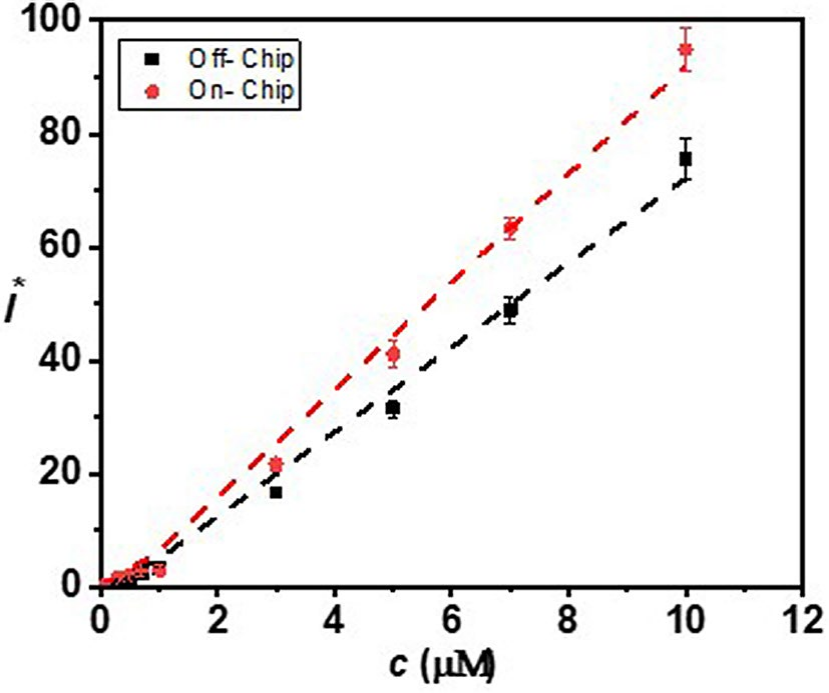
Variation of FL intensity with ${\mathrm{H}}_{2}{\mathrm{O}}_{2}$ concentration in buffer measured using the microfluidic device with on-chip and off-chip mixing/incubation, linear fits are obtained with R^2^ = 0.99 and 0.98, respectively. Each data point represents the standard deviation (SD) from three different readings.

Next, we demonstrate the assay for the measurement of ${\mathrm{H}}_{2}{\mathrm{O}}_{2}$ using the proposed microfluidic device. For measuring the concentration of ${\mathrm{H}}_{2}{\mathrm{O}}_{2}$ in the buffer in the microfluidic device, ${\mathrm{H}}_{2}{\mathrm{O}}_{2}$ stock at a flow rate 0–2.8 μL min^−1^ and buffer at a flow rate of 3.8–1.0 μL min^−1^ are infused into the device to achieve ${\mathrm{H}}_{2}{\mathrm{O}}_{2}$ concentration in the range of 0–7 µM (see Table S1). In the first case, the buffer containing ${\mathrm{H}}_{2}{\mathrm{O}}_{2}$ at a flow rate of 3.8 μL min^−1^ is mixed and incubated with the probe infused into the device at a fixed flow rate 0.2 μL min^−1^ in the serpentine channel for 15 min before passing to the detection module. In the second case, the ${\mathrm{H}}_{2}{\mathrm{O}}_{2}$ stock, buffer, and probe are mixed and incubated outside the device, thus allowing off-chip mixing and incubation, for 15 min and the mixture is infused into the optofluidic module at a flow rate 4 μL min^−1^. For both cases, the variations of normalized FL intensity with concentrations of ${\mathrm{H}}_{2}{\mathrm{O}}_{2}$ is presented in Fig. 2, which show a linear trend, ${\mathrm{I}}^{*}=\mathrm{A c}$, with A = 9.03 and 7.09 and R^2^ = 0.99 and 0.98 for on-chip and off-chip mixing, respectively. The slope of the line for the off-chip mixing/incubation case is found to be smaller compared to that for the on-chip mixing case which can be attributed to the time delay of ~ 5 min between the completion of off-chip mixing/incubation and on-chip detection. For the on-chip mixing/incubation case, this time delay is ~ 500 ms, which is negligible. Further, since ${\mathrm{H}}_{2}{\mathrm{O}}_{2}$ decomposes after exposure to light and air, in the case of on-chip mixing and incubation, the mixture is minimally exposed to air and light and therefore provides an improved signal. The above experiment verifies that 15 min of incubation time is adequate for the accurate measurement of ${\mathrm{H}}_{2}{\mathrm{O}}_{2}$.

### Factors affecting the measurement of ${\mathbf{H}}_{2}{\mathbf{O}}_{2}$ in blood: blood-plasma separation, deproteinization, and plasma dilution

**Figure 3:**
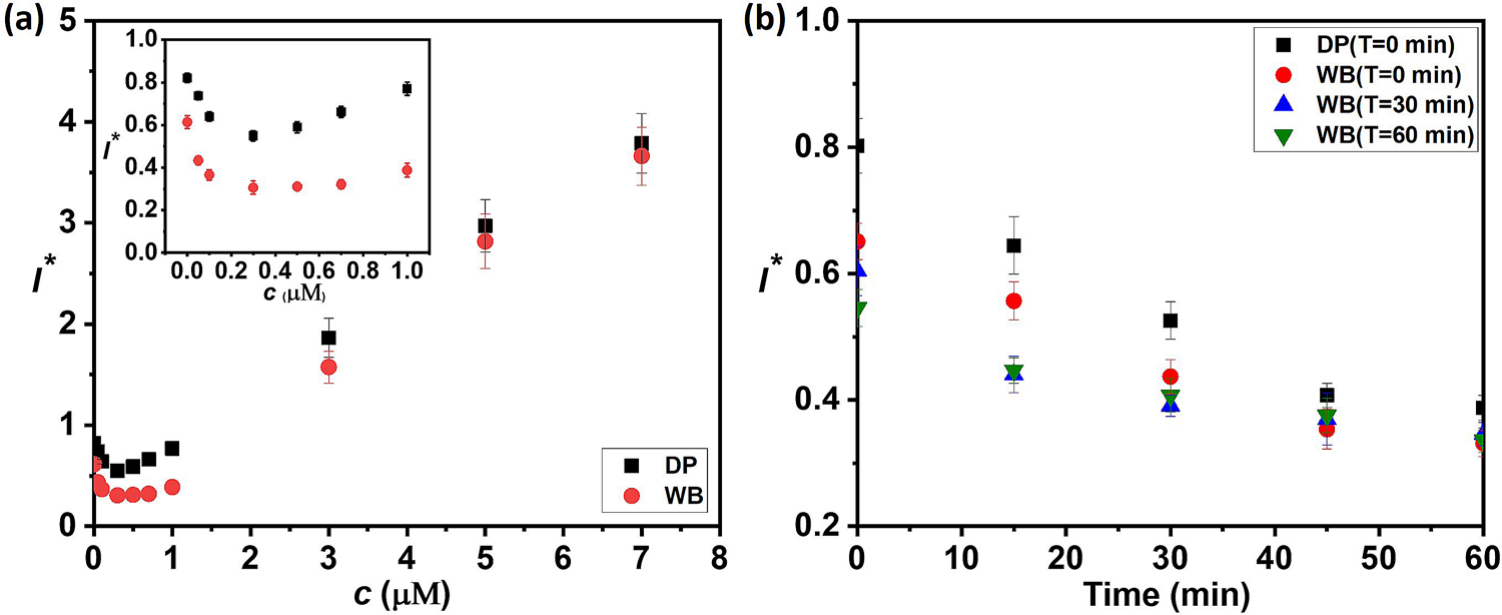
(**a**) Variation of FL intensity with ${\mathrm{H}}_{2}{\mathrm{O}}_{2}$ concentration measured using the microfluidic device, in human whole and deproteinized plasma. (**b**) Variation in FL intensity with measurement time points (t) for whole plasma, after the plasma gets separated at different time points (T) and deproteinized plasma (with T = 0). In all cases centrifuged plasma and on-chip mixing/incubation are used, here DP—deproteinised plasma, and WB—whole blood plasma. (Each data point represents the standard deviation (SD) from three different readings).

Next, we study the effect of blood cells and plasma proteins that alter the native as well as an exogenous concentration of ${\mathrm{H}}_{2}{\mathrm{O}}_{2}$ in blood and thus affect the measurement results and demonstrate plasma dilution as a potential solution to inhibit this effect. First exogenous ${\mathrm{H}}_{2}{\mathrm{O}}_{2}$ in blood plasma obtained via centrifugation is measured using the microfluidic device.${\mathrm{H}}_{2}{\mathrm{O}}_{2}$ stock at a flow rate 0–2.8 μL min^−1^, buffer at a flow rate 3.26–0.46 μL min^−1^$,$ and plasma at a fixed flow rate of 0.54 μL min^−1^ are infused into the device to achieve external ${\mathrm{H}}_{2}{\mathrm{O}}_{2}$ concentration $(\mathrm{c})$ in the range of 0–7 µM (see Table S1). The exogenous ${\mathrm{H}}_{2}{\mathrm{O}}_{2}$ spiked plasma is then mixed and incubated with the probe infused into the device at a fixed flow rate of 0.2 μL min^−1^ in the serpentine channel for 15 min before passing into the detection module. Figure 3a shows the variation of FL intensity with the concentration of exogenous ${\mathrm{H}}_{2}{\mathrm{O}}_{2}$ in plasma. Unexpectedly, there is a fall in the FL intensity with an increase in ${\mathrm{H}}_{2}{\mathrm{O}}_{2}$ concentration initially up to $\mathrm{c}\approx 0.4$ μM (see inset of Fig. 3a) and beyond this concentration, FL intensity increases linearly with increase in concentration (with ${\mathrm{R}}^{2}=0.95$). The initial decrement in the signal with an increase in the ${\mathrm{H}}_{2}{\mathrm{O}}_{2}$ concentration can be attributed to the interaction of blood cells (platelets), proteins, and other plasma constituents such as NOXs and xanthine oxidase with ${\mathrm{H}}_{2}{\mathrm{O}}_{2}$, as discussed elsewhere^1^.

Here, we study the influence of blood cells and larger plasma proteins on the dynamic change of endogenous ${\mathrm{H}}_{2}{\mathrm{O}}_{2}$. We also explore the dilution of plasma to minimize the interference due to the cells, proteins, and other plasma constituents. Blood samples collected from healthy individuals at a fixed time point were centrifuged at different time points ($\mathrm{T}$ = 0 min, 30 min, and 60 min) allowing interaction of the endogenous ${\mathrm{H}}_{2}{\mathrm{O}}_{2}$ with the blood cells for different time durations. The plasma obtained from centrifugation of blood samples at these different time points after collection is measured at varying time points $(\mathrm{t})$ after centrifugation, every 15 min. The variation of FL intensity of endogenous ${\mathrm{H}}_{2}{\mathrm{O}}_{2}$ with time is presented in Fig. 3b. It is observed that for a given centrifuged plasma sample, the FL intensity decreases with time suggesting endogenous ${\mathrm{H}}_{2}{\mathrm{O}}_{2}$ concentration in the plasma samples decreases with time. This can be because, in in-vitro condition, blood plasma does not interact with the endothelium and the tissues and therefore blood is devoid of production or the supply of ${\mathrm{H}}_{2}{\mathrm{O}}_{2}$ contributed by NOXs (nicotinamide adenine dinucleotide phosphate oxidase) on the surface of phagocytes and endothelial cells and xanthine oxidase bound to endothelial cells^1^. Further, plasma proteins act as a sink and consume ${\mathrm{H}}_{2}{\mathrm{O}}_{2}$ leading to a decrease in endogenous ${\mathrm{H}}_{2}{\mathrm{O}}_{2}$ with time. The FL intensity of plasma centrifuged at $\mathrm{T}=0$ is higher than that of plasma obtained from centrifugation of blood sample at T = 30 min and 60 min (Fig. 3b), suggesting that in the in-vitro condition the blood cells act as a sink for ${\mathrm{H}}_{2}{\mathrm{O}}_{2}$. Literature reports that blood cells including red blood cells can exchange ${\mathrm{H}}_{2}{\mathrm{O}}_{2}$ from multiple sources although the level of intracellular ${\mathrm{H}}_{2}{\mathrm{O}}_{2}$ is maintained due to the catalase and peroxidases^1^. Therefore, besides preventing interference with optical measurements, separation of plasma from whole blood becomes necessary to eliminate the effect of blood cells on the dynamic change of ${\mathrm{H}}_{2}{\mathrm{O}}_{2}$ for accurate measurement of ${\mathrm{H}}_{2}{\mathrm{O}}_{2}$.

To minimize the effect of plasma proteins on the consumption and therefore the dynamic change of ${\mathrm{H}}_{2}{\mathrm{O}}_{2}$, immediately after sample collection and centrifugation, the centrifuged plasma is deproteinized using a 10kD filtration column. A comparison of the FL intensity levels with time measured for whole plasma and deproteinized plasma is shown in Fig. 3b. The results show that deproteinization significantly improves the FL intensity indicating that the reaction between ${\mathrm{H}}_{2}{\mathrm{O}}_{2}$ and the probe becomes more efficient in the absence of larger proteins (> 10 kD). This can indicate that possibly larger proteins tend to quench the effect of the probe^30^. However, the FL intensity continues to decrease with time suggesting that smaller proteins (< 10 kD) contribute towards the consumption of plasma ${\mathrm{H}}_{2}{\mathrm{O}}_{2}$. A higher slope of the curve for the deproteinized plasma indicates that smaller proteins consume ${\mathrm{H}}_{2}{\mathrm{O}}_{2}$ faster leading to faster degradation in the FL intensity in the absence of larger proteins. To study the effect of exogenous ${\mathrm{H}}_{2}{\mathrm{O}}_{2}$ in deproteinized plasma, the different concentration of exogenous ${\mathrm{H}}_{2}{\mathrm{O}}_{2}$ in deproteinized plasma is measured and the variation of FL intensity with ${\mathrm{H}}_{2}{\mathrm{O}}_{2}$ concentration is shown in Fig. 3a. Although the FL intensity for deproteinized plasma is higher, in both cases, an initial decrement in FL intensity is observed up to a concentration of 0.4 µM. Therefore, although the removal of larger proteins leads to a higher FL intensity attributed to a more efficient ${\mathrm{H}}_{2}{\mathrm{O}}_{2}$ and probe reaction, the consumption of plasma ${\mathrm{H}}_{2}{\mathrm{O}}_{2}$ and consequently, the decrease in the FL intensity with time is caused by the smaller proteins. The removal of smaller proteins has been demonstrated^31^ but such methods use precipitants such as Trichloroacetic acid (TCA), which can alter the native ${\mathrm{H}}_{2}{\mathrm{O}}_{2}$ concentration and therefore we do not explore such methods. Instead, we explore the dilution of plasma to minimize the interference of smaller proteins and overcome decrement in FL intensity at smaller concentrations.

**Figure 4:**
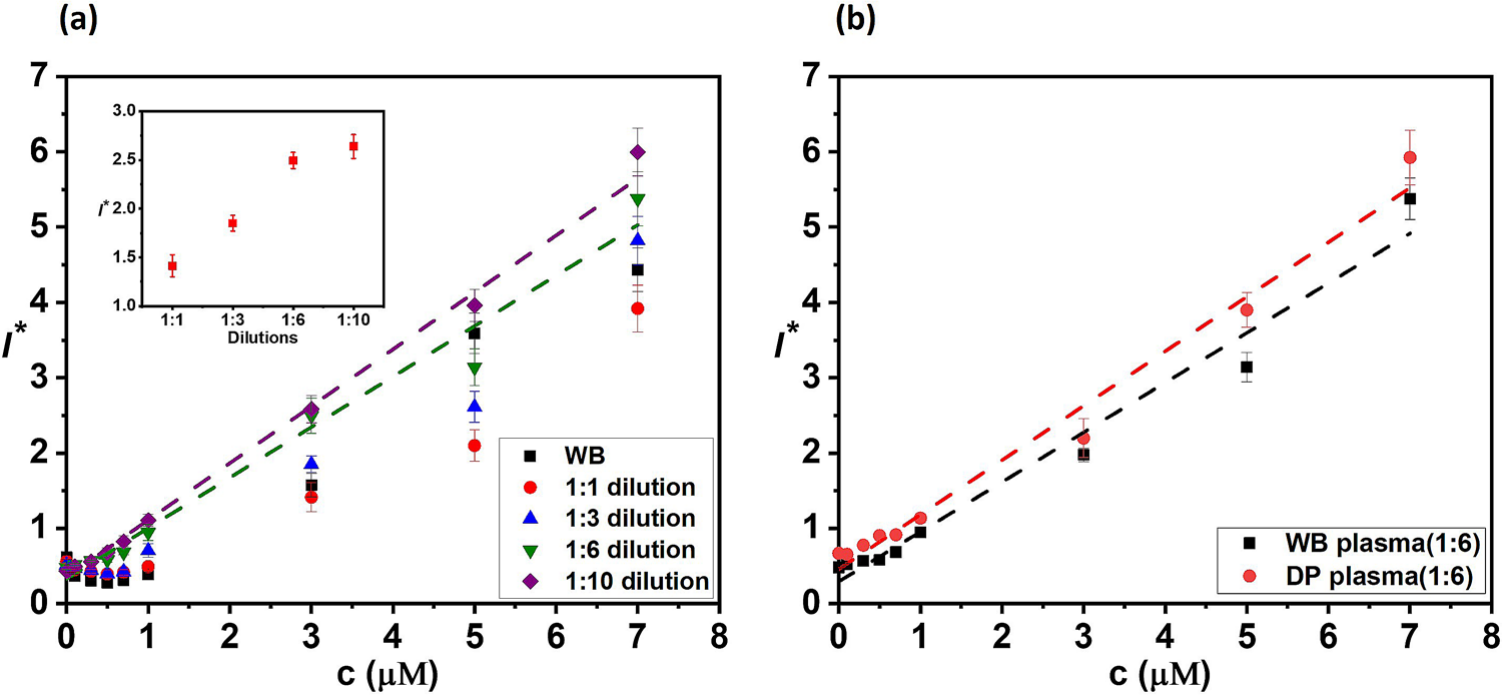
(**a**) Variation of FL intensity with ${\mathrm{H}}_{2}{\mathrm{O}}_{2}$ concentration in buffer-diluted whole human plasma at different dilutions, inset shows the variation of FL intensity with dilution for $c=3$ (linear fits with ${\mathrm{R}}^{2}=0.98$ for both 1:6 and 1:10 dilutions). (**b**) Variation of FL intensity with ${\mathrm{H}}_{2}{\mathrm{O}}_{2}$ concentration in buffer-diluted deproteinized and whole human plasma at 1:6 dilution (DP—deproteinised plasma, and WB—whole blood plasma). (Each data point represents the standard deviation (SD) from three different readings).

We performed experiments to study the effect of plasma dilution on the reduced interaction between plasma proteins and ${\mathrm{H}}_{2}{\mathrm{O}}_{2}$. Immediately after sample collection and centrifugation ($\mathrm{at T}=0$), the centrifuged plasma is diluted with buffer at different dilutions in the range 1:1–1:10 (see flow rates in Table S3 in “Supplementary Material”). Figure 4a shows the variation of the measured FL intensity with the concentration of exogenous ${\mathrm{H}}_{2}{\mathrm{O}}_{2}$ in the diluted plasma at different dilutions. Interestingly, we see that the initial decrement in FL intensity with an increase in the exogenous ${\mathrm{H}}_{2}{\mathrm{O}}_{2}$ concentration is not observed at higher dilutions indicating that the interference due to plasma proteins and other molecules is suppressed. The results show that at a dilution of 1:6, the interference is eliminated and a linear increase in the FL intensity with the exogenous plasma concentration is observed, and ${I}^{*}=0.67 c+0.33$ (with ${\mathrm{R}}^{2}=0.98$). The inset in Fig. 4a (at $c=3$ μM) shows that FL intensity increases with an increase in dilution due to a decrease in interference due to proteins and other elements. Since we have achieved linearity with 1:6 dilutions and there is no significant change in intensity between 1:6 and 1:10 dilutions, we proceed with 1:6 dilutions. The variation of FL intensity with ${\mathrm{H}}_{2}{\mathrm{O}}_{2}$ concentration for deproteinized plasma after removal of larger proteins (> 10 kD) and whole plasma at 1:6 dilution presented in Fig. 4b shows that deproteinization improves the FL intensity but linearity is observed in both cases. However, the implementation of on-chip deproteinization requires special techniques such as electrophoresis^32^ or iso-tachophoresis^15^ which complicates the device fabrication and operation. Therefore, we proceed with 1:6 dilutions that suppresses the interference due to proteins and other small molecules, in the subsequent studies.

### Measurement of ${\mathbf{H}}_{2}{\mathbf{O}}_{2}$ in blood plasma using the microfluidic device

**Figure 5:**
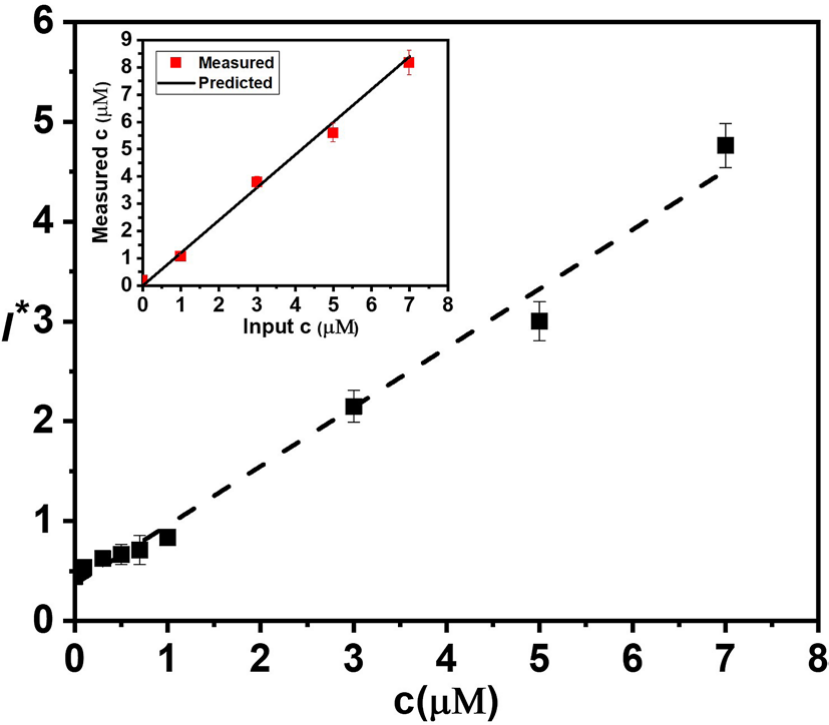
Variation of FL intensity with ${\mathrm{H}}_{2}{\mathrm{O}}_{2}$ concentration in human whole plasma at 1:6 dilution, measured using the integrated microfluidic device with on-chip blood plasma separation and on-chip mixing/incubation), a linear fit is obtained with ${\mathrm{R}}^{2}=0.99$. Inset shows a comparison of the input ${\mathrm{H}}_{2}{\mathrm{O}}_{2}$ concentrations and predicted ${\mathrm{H}}_{2}{\mathrm{O}}_{2}$ concentrations from FL measurements, which also shows a linear fit with ${\mathrm{R}}^{2}=0.98$. (Each data point represents the standard deviation (SD) from three different readings).

Finally, the integrated device, comprising blood plasma separation, mixing and incubation, and optical detection modules, is used to measure the actual quantities of exogenous ${\mathrm{H}}_{2}{\mathrm{O}}_{2}$ in blood plasma obtained on-chip at different concentrations and predict endogenous ${\mathrm{H}}_{2}{\mathrm{O}}_{2}$ concentration in healthy individuals. Whole blood samples collected from healthy volunteers are diluted with buffer at a 1:2 dilution ratio and infused into the microchannel at a flow rate of 20 μL min^−1^ to obtain a plasma flow rate of 1.0 μL min^−1^. The remaining four-fold dilution of the plasma is obtained on-chip by maintaining the flow rates shown in Table S1 to achieve a final 1:6 dilution. The ${\mathrm{H}}_{2}{\mathrm{O}}_{2}$ stock at a flow rate 0–2.8 μL min^−1^, buffer at a flow rate 2.8–0 μL min^−1^$,$ and plasma at a fixed flow rate of 1.0 μL min^−1^ was infused into the device to obtain exogenous ${\mathrm{H}}_{2}{\mathrm{O}}_{2}$ concentration in the range of 0–7 µM. The ${\mathrm{H}}_{2}{\mathrm{O}}_{2}$ in plasma is then mixed and incubated with the probe infused into the device at a fixed flow rate of 0.2 μL min^−1^ in the serpentine channel for 15 min before entering the detection module. The variation of FL intensity with the concentration of exogenous ${\mathrm{H}}_{2}{\mathrm{O}}_{2}$ in plasma measured using the integrated device is depicted in Fig. 5. A linear increase in the FL intensity with the plasma ${\mathrm{H}}_{2}{\mathrm{O}}_{2}$ concentration is observed, ${I}^{*}=0.60c+0.37$ (with ${\mathrm{R}}^{2}=0.98$). The calibration obtained from the data presented in Fig. 4b is used to predict the concentration of ${\mathrm{H}}_{2}{\mathrm{O}}_{2}$ in plasma and is compared against the actual exogenous concentration of ${\mathrm{H}}_{2}{\mathrm{O}}_{2}$, as shown in the inset of Fig. 5. The curve is linear with a slope $\approx 1$ and a very good match (within 2%) between the predicted and actual ${\mathrm{H}}_{2}{\mathrm{O}}_{2}$ concentration is obtained indicating that the integrated device can accurately measure the concentration of ${\mathrm{H}}_{2}{\mathrm{O}}_{2}$ in plasma. Considering the 1:6 dilution, the limit of detection of the device and assay is found to be 0.05 µM, and sensitivity is found to be 0.60 µM^−1^ (See the Section S5 in “Supplementary Material” and Fig. S4 for a zoomed view in the concentration range 0–1 µM).

We collected whole blood samples from ten healthy individuals and measured the range of the values of ${I}^{*}$. For the exogenous ${\mathrm{H}}_{2}{\mathrm{O}}_{2}$ concentration $c=0$, we obtained I^*^ = 0.37–0.8, which, upon considering the dilutions, predicts the endogenous concentration in the range 0.8–6 µM. A detailed comparison of the different existing methods and the present work is presented in Table 1. Most of the existing methods employ the electrochemical technique for the detection of ${\mathrm{H}}_{2}{\mathrm{O}}_{2}$. The main contribution of the present work is that we demonstrate an automated device for direct and continuous monitoring of ${H}_{2}{O}_{2}$ in human blood samples on-chip, and report the endogenous concentration of ${H}_{2}{O}_{2}$ in blood plasma. Further, most of the existing methods demonstrate ${H}_{2}{O}_{2}$ measurement in buffer, serum, or urine and direct measurement of ${H}_{2}{O}_{2}$ from the blood sample is not available. Further, externally added ${H}_{2}{O}_{2}$ in serum separated from blood manually is measured using the electrochemical method that involves manual steps^22,24^, and measurements of endogenous ${H}_{2}{O}_{2}$ is not available. Also, a major disadvantage of the electrochemical method is the fouling of electrodes when used with biological samples, which reduces accuracy and sensitivity of measurement with time. Further, many electrochemical methods involved immobilisation of enzymes on the electrode surface, which can interfere with the analyte and affect the measurement. On the other hand, in optical-based detection, the sensitivity of the system remains unchanged and the device can be used over a long time without loss of accuracy or sensitivity. The only disadvantage of this system compared with electrochemical detection is the cost aspect which mainly arises because of the expensive laser and multiple syringe pumps used. But, the cost of the laser and syringe pumps can be reduced by adapting to low-cost LED sources and peristaltic pumps, respectively that could pave the way for the development of a point-of-care device. The microfluidic device and method proposed here can be used for the measurement of ${\mathrm{H}}_{2}{\mathrm{O}}_{2}$ from a minimum of 200 µL of blood within 15 min, for clinical applications. The device also holds great promise for real-time measurement of ${\mathrm{H}}_{2}{\mathrm{O}}_{2}$ in ICU patient's blood for prediction of system inflammatory syndrome (SIRS) and other medical emergency conditions^1^.

**Table 1:** A compariso of the fluorometric method used here with previously reported electrochemical methods.

Method of detection	Sensitivity	Linearity range	LOD	Response time	Sample used	Refs.
Electrochemical	39.2 μA mM^−1^ cm^−2^	0.5 μM–50 mM	60 nM	2.0 s	Human Urine	^21^
Electrochemical	662.6 μA mM^−1^ cm^−2^	0.3–704.8 μM	30 nM	2.0 s	Human blood serum	^22^
Electrochemical		10 nM–1 μM	10 nM		Buyer	^23^
Electrochemical	0.103 μA log(M) cm^−2^	0.1 pM–0.1 μM	0.1 pM	6.0 s	Human blood serum	^24^
Electrochemical	1.46 nA nM^−1^ cm^−2^	10 nM–1 μM	5 nM		Buyer	^27^
Fluorometric	0.6 μM^−1^	50 nM–49 μM	50 nM	Instantaneous	Human blood plasma	Present work

## Conclusions

We reported a method for direct and rapid measurement of ${\mathrm{H}}_{2}{\mathrm{O}}_{2}$ in human whole blood by employing a microfluidic device comprising a blood plasma separation module and a mixing and incubation module. The critical factors that affect the dynamics of ${\mathrm{H}}_{2}{\mathrm{O}}_{2}$ in the blood sample and therefore significantly influence the measurement accuracy was identified—it was found that the presence of blood cells and soluble proteins can significantly alter the native ${\mathrm{H}}_{2}{\mathrm{O}}_{2}$ levels in the time interval between sample withdrawal and detection. The removal of larger proteins (> 10 kDa) improved the signal but did not eliminate the interference of plasma proteins with ${\mathrm{H}}_{2}{\mathrm{O}}_{2}$, depicting the role of smaller proteins. Our study revealed that separation of blood cells and subsequent dilution of the cell-free plasma with buffer at a dilution ratio of 1:6 inhibit the interference effect. While on-chip deproteinization will require more complex techniques, on-chip blood plasma separation and dilution could be easily implemented. Our method was used to demonstrate rapid measurement of ${\mathrm{H}}_{2}{\mathrm{O}}_{2}$ in blood plasma in the concentration range of 0–49 µM, with a limit of detection of 0.05 µM, a sensitivity of 0.6 µM^−1^, and can facilitate both discrete and real-time and continuous measurement of ${\mathrm{H}}_{2}{\mathrm{O}}_{2}$ in the patient’s blood every 15 min, requiring a minimum of 300 µL of blood for discrete measurements. Using the linear correlation, ${I}^{*}=0.60c+0.37$ (with ${R}^{2}>0.98$) developed with exogenous ${\mathrm{H}}_{2}{\mathrm{O}}_{2}$, and considering dilutions, the concentration of endogenous ${\mathrm{H}}_{2}{\mathrm{O}}_{2}$ in the blood of healthy individuals was predicted to be in the range 0.8–6 µM. The measurement time of 15 min is mainly due to the time required to complete the reaction between ${\mathrm{H}}_{2}{\mathrm{O}}_{2}$ and the probe. If a fluorescence probe with a faster reaction rate is discovered in the future, and the assay can be performed in a shorter time, that will certainly reduce the measurement time. Herein, a proof of concept of a lab-scale microfluidic system has been demonstrated, that can be further developed into a point-of-care system. The system can be simplified—for example, the syringe pumps and laser can be replaced by small and cost-effective peristaltic pumps and LED sources, to develop a point of care device for use at the bedside. Our method and device can be used for accurate measurement of ${\mathrm{H}}_{2}{\mathrm{O}}_{2}$, an important signalling molecule, and an early indicator of oxidative stress. The availability of this device at the point of care will significantly help in understanding the role of ${\mathrm{H}}_{2}{\mathrm{O}}_{2}$ in health and disease.

## Materials and methods

Phosphate Buffered Saline (PBS) of concentration 0.01 M and pH 7.4 is prepared by dissolving PBS (Sigma-Aldrich, USA) in DI water. Blood samples are collected from the healthy volunteers in heparin-coated vacutainers (All methods were carried out following relevant guidelines and regulations. All experimental protocols were approved by the Institute Ethics Committee, IIT Madras (Ref. No. IEC/2020/02/AK-1/01). Informed consent was obtained from all subjects. All were adults and consented themselves). For experiments with centrifuged plasma, the collected blood sample is immediately centrifuged for 10 min at 6000 rpm and the plasma is collected (Fig. S1b). For experiments with deproteinized plasma, the plasma is centrifuged through 10 kD filtration columns (abcam, USA) at a speed of 10,000 × *g* for 10 min to remove the proteins > 10 kD. For experiments with on-chip separated plasma, the whole blood sample diluted with PBS (1:2 dilution) is used.

Figure S2c shows that the plasma separated on-chip has higher purity, as it shows a lower absorbance compared to the centrifuged plasma. In all cases, the volume of sample blood is 200 µL, which is sufficient to maintain a continuous flow in the serpentine channel for 15 min. A mixture of amplex red substrate and horseradish peroxidase (HRP) (Fluorimetric Hydrogen Peroxide Assay Kit, Sigma Aldrich, USA) is used as the probe for the detection of ${\mathrm{H}}_{2}{\mathrm{O}}_{2}$ in buffer and plasma. The HRP catalyzes the reduction of ${\mathrm{H}}_{2}{\mathrm{O}}_{2}$ to water and in the presence of amplex red, which acts as the hydrogen donor, HRP reacts with ${\mathrm{H}}_{2}{\mathrm{O}}_{2}$ in a 1:1 stoichiometry ratio to produce resorufin, which is a fluorescent compound. First, the assay is tested via fluorescence-based measurement of ${\mathrm{H}}_{2}{\mathrm{O}}_{2}$ in the buffer in a 96-well plate reader (LS-55, Perkin Elmer Inc., USA) and subsequently using the microfluidic device. Then, externally added (exogenous) ${\mathrm{H}}_{2}{\mathrm{O}}_{2}$ at different concentrations in blood plasma obtained via centrifugation is measured using the 96-well plate reader and the microfluidic device. Further, the effect of the removal of blood cells and plasma proteins, and dilutions with buffer on the ${\mathrm{H}}_{2}{\mathrm{O}}_{2}$ measurement is studied. Finally, experiments are performed for the direct measurement of endogenous ${\mathrm{H}}_{2}{\mathrm{O}}_{2}$ in sample blood infused into the device.

The normalised fluorescence intensity is calculated using the equation, ${I}^{*}=\left(I-{I}_{o}\right)/{I}_{en} ,$ where $I$ is the FL intensity measured at a particular exogenous concentration of ${H}_{2}{O}_{2}$, and ${I}_{o}$ is the FL intensity of only the probe in buffer without any ${H}_{2}{O}_{2}$ and ${I}_{en}$ is the FL intensity due to endogenous ${H}_{2}{O}_{2}$ (i.e. probe + plasma and no exogenous ${H}_{2}{O}_{2}$). Therefore, ${I}^{*}$ accounts for both the endogenous and exogenous ${H}_{2}{O}_{2}$ concentrations. To obtain the calibration curve, we collected blood samples from three healthy individuals and extracted the plasma, and diluted the plasma with buffer at 1:6 to reduce the interference of plasma proteins on the ${H}_{2}{O}_{2}$ measurement. After dilution, we first measure the FL intensity corresponding to the endogenous concentration (i.e. ${I}_{en}$). Subsequently, we measure $I$ at different exogenous concentrations of ${H}_{2}{O}_{2}$, and calculate the normalized FL intensity ${I}^{*}$ at each exogenous ${H}_{2}{O}_{2}$ concentration, as shown in Figs. 4b and 5. In Fig. 4b, the plasma is obtained from centrifugation, and in Fig. 5, the plasma is separated on-chip using the acoustics device. Next, to determine the endogenous ${H}_{2}{O}_{2}$ concentration in healthy individuals, we obtained the blood samples of ten healthy individuals, separated plasma on-chip, and diluted at 1:6. We measured the endogenous FL intensity, ${I}_{en}$ for each of the ten samples and used the correlation obtained from Fig. 5 and considered the dilution factor to predict the average endogenous concentration. Each data point in all the results is obtained after performing three sets of experiments and in each individual experiment, the readings are the average of five different readings.

### Ethics declarations

All methods were carried out following relevant guidelines and regulations. All experimental protocols were approved by the Institute Ethics Committee, IIT Madras (Ref. No. IEC/2020/02/AK-1/01). Informed consent was obtained from all subjects or if subjects are under 18, from a parent and/or legal guardian.

## Supplementary Information

Supplementary Information.

## Data Availability

The datasets generated during and/or analysed during the current study are available from the corresponding author on reasonable request.
