# Supplementary material for: Direct and rapid measurement of hydrogen peroxide in human blood using a microfluidic device
Source: Sci Rep. 2021 Feb 3;11:2960. doi: 10.1038/s41598-021-82623-4 (PMC7858642; doi:10.1038/s41598-021-82623-4)
Supplement: Supplementary file 1 — Supplementary Information. [file 41598_2021_82623_MOESM1_ESM.pdf]

Supplementary Information

Direct and rapid measurement of hydrogen peroxide in human blood using a microfluidic device

R. Gaikwad[1], P. R. Thangaraj[2], and A. K. Sen[1,*]

[1]Micro Nano Bio -Fluidics Unit, Fluid Systems Laboratory, Department of Mechanical Engineering, Indian Institute of Technology Madras, Chennai-600036, India.
[2]Department of Cardiothoracic Surgery, Apollo Hospital, Chennai, 600006, India.

[*]Corresponding author: ashis@iitm.ac.in

Table of contents-

- Acoustics-based for blood-plasma separation.
- Flow rates of the buffer, H_2O_2 stock, plasma, and probe for on-chip mixing and reaction and detection of exogenous H_2O_2 in the buffer and blood plasma, centrifugation and on-chip, at different concentrations for 1:6 dilution.
- Optical transmission of machined, chloroform exposed, and heat-treated PMMA channel.
- Fluorescence imaging inside microchannel and in Eppendorf tubes.
- Schematic of the experimental setup, Blood plasma separation- images and absorbance measurement, FL signal from H_2O_2+probe mixture in Eppendorf tube.
- Variation of FL intensity with H_2O_2 concentration in buffer measured using the microfluidic device with on-chip and off-chip mixing/incubation.
- Flow rates of the buffer, H_2O_2 stock, plasma, and probe for on-chip mixing and incubation and detection of exogenous H_2O_2 in the centrifuged blood plasma at different concentrations and for 1:1, 1:3, and 1:10 dilutions.
- Limit of detection and sensitivity.

S1: Acoustics-based for blood-plasma separation

The blood plasma separation device works on the principle of acoustophoresis. The blood sample flowing through the microchannel is exposed to bulk acoustic standing waves generated using a PZT resonator. The width of the microchannel is 300 µm and by operating the PZT at a frequency of 1.91 MHz, standing half-waves are produced with the node at the center of the microchannel and anti-nodes at the side walls. Micron-sized objects exposed to standing bulk acoustic wave can experience primary acoustic radiation force given by[25] $F_p = 4\pi a^3 E_{ac} k \sin(2kz)\phi$, with $\phi = \frac{\rho_p + \frac{2}{3}(\rho_p - \rho_0)}{2\rho_p + \rho_0} - \frac{1}{3}\frac{\beta_p}{\beta_0}$, $\beta_0 = \frac{1}{\rho_0 c_0^2}$, $\beta_p = \frac{1}{\rho_p c_p^2}$, $E_{ac} = \frac{P_a}{4\rho_0 c_0^2}$, where a is the particle size, E_{ac} is the acoustic energy density, k is the wavenumber, z is the distance from the wall, ϕ is the contrast factor, ρ_p and ρ_0 are the density of the particles and the density of the medium respectively, β_p and β_0 are the compressibility of the particles, and the medium respectively, c_0 and c_p is the velocity of sound in the medium and particle respectively, and the acoustic-pressure amplitude is P_a. The blood cells exposed to the standing waves experience the acoustic radiation force due to a higher acoustic impedance compared to the suspending medium or the plasma, consequently a positive contrast factor and therefore migrate towards the nodal plane. The blood cells get focused at the center of the microchannel and exit through the center outlet whereas the cell-free plasma enters into the mixing and incubation module through the side outlets. In the present study, the flow rate of the whole blood sample is kept fixed at 20 µL/min and acoustic energy density is also kept fixed 14.9 J/m^3 to obtain cell-free plasma at a flow rate of 1.0 µL/min."

S2: The details of flow rates for different H_2O_2 concentrations in various experiments

Here, the H_2O_2 stock of 10 µM concentration, used as a working solution, is filled in one of the syringes, and the other syringes are separately filled with the probe, buffer, and plasma. The flow rates of probe, buffer, and plasma are adjusted to achieve a total flow rate of 4 µL/min. All the flow rates, such as the H_2O_2 stock, buffer, probe, and plasma, are adjusted considering the final flow rate of 4 µL/min.

For example, in experiments with buffer (see Table S1), to obtain the final H_2O_2 concentration of 1.0 µM, the flow rates are 3.4 µL/min buffer, 0.4 µL/min H_2O_2 stock, and 0.2µL/min probe, which gives a total flow rate of 4 µL/min. By molarity calculation, say per minute (or any fixed time duration), we get the concentration of 1.0 µM in a final volume of 4.0 µL by adding 0.4 µL from the stock of 10 µM. The process is continuous and the other concentration is achieved in the same way.

Similarly, in the experiments with plasma (see Table S1), a similar procedure is followed; the only difference is that the fixed 1:6 plasma dilution (which is required to overcome the interference from plasma proteins) is achieved by fixing the plasma volume and total (buffer + stock) volume. The individual buffer and stock volumes are varied to achieve a given concentration.

To avoid cross-contamination, the channels are flushed with the buffer between the measurements at different concentrations. During this periodic cleaning step, the setup remains fixed and does not require any manual intervention; only the other syringe infusion pumps are turned off leaving the buffer infusion pump running.

Table S1 Flow rates of the buffer, H_2O_2 stock, plasma and probe for on-chip mixing (and incubation) and detection of exogenous H_2O_2 in the buffer and blood plasma (centrifugation and on-chip) at different concentrations for 1:6 dilution.

H_2O_2 concentration (μM)	H_2O_2 stock $(\mu L/min)$	*Experiments with buffer*	*Experiments with centrifuged plasma*		*Experiments with on-chip separated plasma (1:6)*		Probe $(\mu L/min)$
		Buffer (μL/min)	Buffer $(\mu L/min)$	Plasma $(\mu L/min)$	Buffer $(\mu L/min)$	Plasma $(\mu L/min)$	
0	0	3.80	3.26	0.54	2.80	1.0	0.2
0.1	0.04	3.76	3.22	0.54	2.76	1.0	0.2
0.3	0.12	3.68	3.14	0.54	2.68	1.0	0.2
0.5	0.20	3.60	3.06	0.54	2.60	1.0	0.2
0.7	0.28	3.52	2.98	0.54	2.52	1.0	0.2
1.0	0.40	3.40	2.86	0.54	2.40	1.0	0.2
3.0	1.20	2.60	2.06	0.54	1.60	1.0	0.2
5.0	2.00	1.80	1.26	0.54	0.80	1.0	0.2
7.0	2.80	1.00	0.46	0.54	0.00	1.0	0.2

S3: Optical transmission of machined, chloroform exposed and heat-treated PMMA channel

The microchannel was machined in PMMA using a CNC micro-milling machine (Minitech machinery, USA) and then exposed to chloroform vapour for 2 min before sealing it with a planar PMMA substrate and then heating the bonded device at 65°C for 30 min. We have measured the roughness of a microchannel machined and exposed to a heat cycle at the same conditions using a surface profiler (Wyko NT1100, Vecco, USA), which was found to be ~30 nm. This is in agreement with the literature which suggests that micro-milled PMMA after exposure to chloroform and a heating cycle (at 60°C) yields optical quality devices with reduced roughness[2]. We have also compared the transmittance of the PMMA microchannel prepared above with that of unprocessed PMMA and glass. The transmittance measurements were performed using a spectrometer (Flame-T, Ocean Optics, Germany) and a light source (DH-2000-BAL, Ocean Optics, Germany). We observed a negligible difference between the transmittance values obtained in the three different cases indicating that the fabrication process does not affect the optical quality of the PMMA considerably (See Fig. S1).

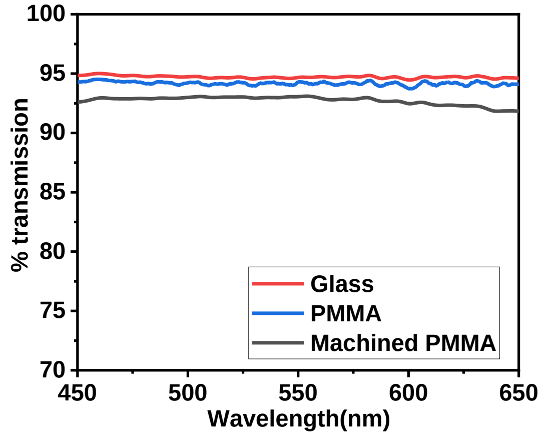

Fig. S1 Percentage of optical transmissions at different frequencies for different surfaces such as glass, PMMA, and machined PMMA with chloroform exposure and heat cycle of 65°C for 30min.

S4: Fluorescence imaging inside microchannel and in Eppendorf tubes

The images in Figure 1c and S2b are the fluorescence images of the mixture of H_2O_2 and the chemical probe upon reaction, for different concentrations of H_2O_2, captured in the microchannels and Eppendorf tubes. The images shown for the microchannel case are captured by passing a laser beam through the channel in the detection module in the dark field and using a 60X lens of an inverted microscope and a high-speed/resolution colour camera. The images for the Eppendorf tube case are captured with an 18MP phone camera, placing the tubes on a UV illuminator. The RGB values for the pictures after subtracting the background are shown in the table below.

Table S2 The RGB values for the fluorescence images after subtracting the background.

	H_2O_2 Concentrations (μM)				
**RGB values**	0	1	3	5	7
Microchannel	(23,8,0)	(30,14,0)	(44,21,0)	(74,32,0)	(101,46,0)
Eppendorf tube	(107,40,38)	(138,53,52)	(175,63,45)	(186,65,42)	(214,73,41)

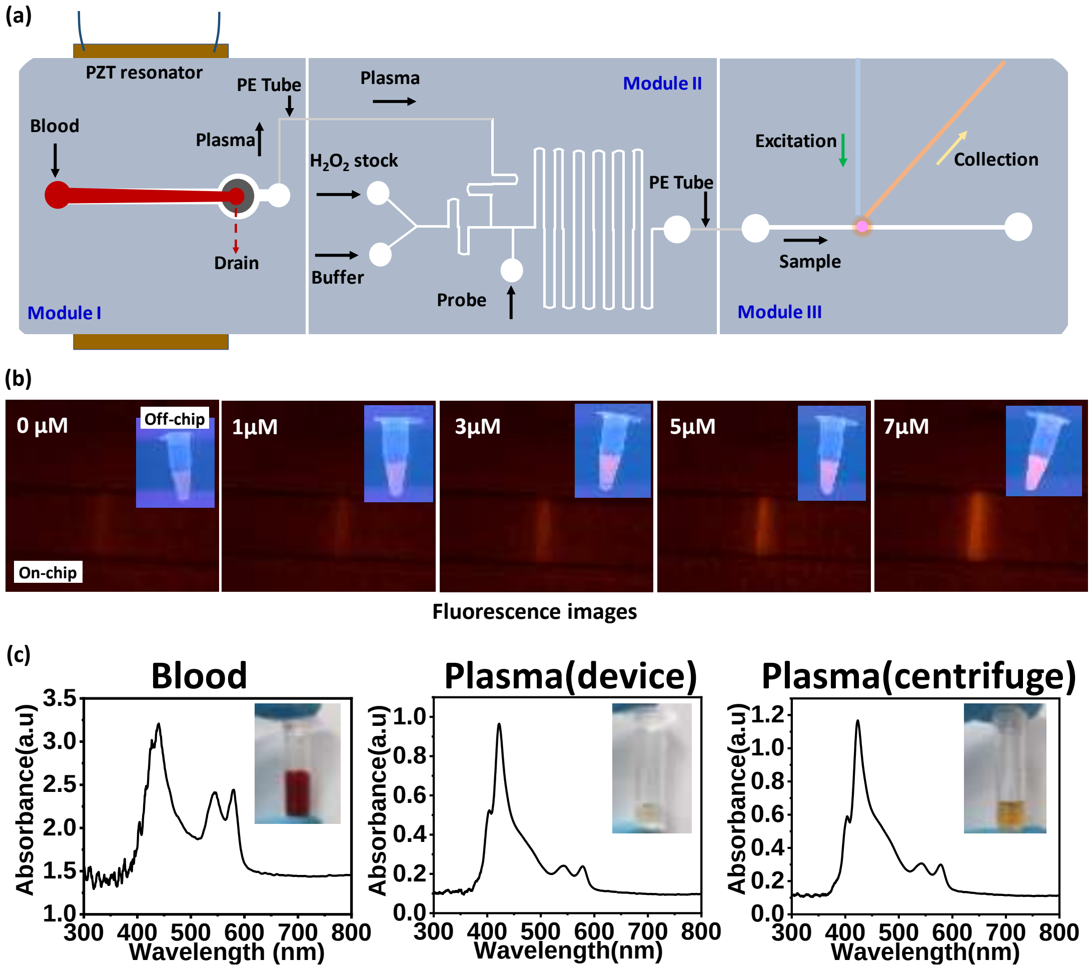

Fig. S2 (a) Schematic of the microfluidics device, (b) FL images of H_2O_2+probe mixture in Eppendorf tube, (c) Blood plasma separation- images and absorbance measurement.

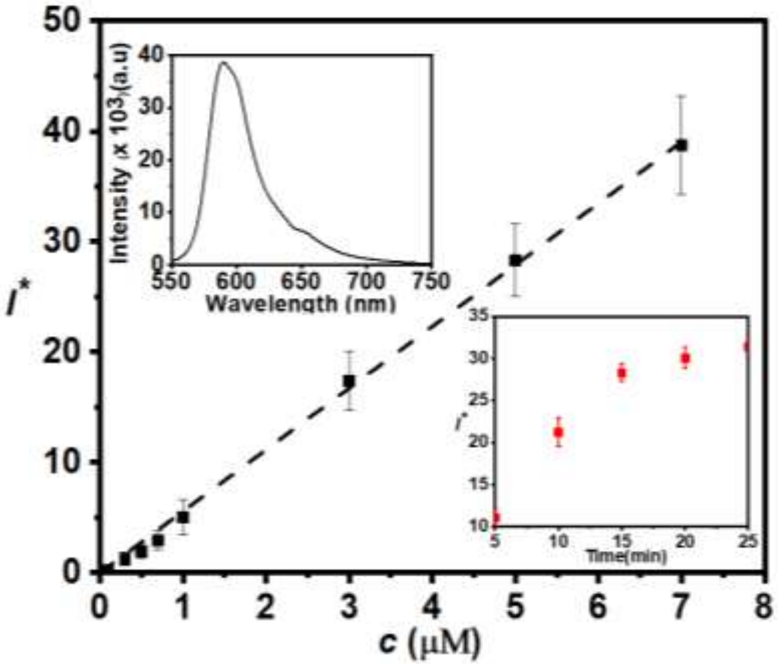

Fig. S3 Variation of FL intensity with H_2O_2 concentration in buffer measured using a 96-well plate reader (data shows a linear fit with $R^2 = 0.98$, and error bar shows the SD from three readings), inset shows the variation in FL intensity with mixing/incubation time at a concentration of 5 μM. Each data point represents the average of five different readings. The error bar represents the standard deviation of those readings. The increase in FL intensity with 20 min incubation compared to 15 min incubation is <5% and hence we proceed with 15 min of incubation time.

Table S3 Flow rates of the buffer, H_2O_2 stock (15 μM), plasma and probe for on-chip mixing and reaction and detection of exogenous H_2O_2 in the centrifuged blood plasma at different concentrations and at different dilutions.

$\mathbf{H_2O_2}$ concentration (**μM**)	$\mathbf{H_2O_2}$ stock (**μL/min**)	*1:1 dilution*		*1:3 dilution*		*1:10 dilution*		Probe (**μL/min**)
		Buffer (**μL/min**)	Plasma (**μL/min**)	Buffer (**μL/min**)	Plasma (**μL/min**)	Buffer (**μL/min**)	Plasma (**μL/min**)	
0	0	1.9	1.9	2.85	0.95	3.45	0.35	0.2
0.1	0.03	1.87	1.9	2.82	0.95	3.42	0.35	0.2
0.3	0.08	1.82	1.9	2.77	0.95	3.37	0.35	0.2
0.5	0.13	1.77	1.9	2.72	0.95	3.32	0.35	0.2
0.7	0.18	1.72	1.9	2.67	0.95	3.27	0.35	0.2
1.0	0.27	1.63	1.9	2.58	0.95	3.18	0.35	0.2
3.0	0.80	1.1	1.9	2.05	0.95	2.65	0.35	0.2
5.0	1.33	0.57	1.9	1.52	0.95	2.12	0.35	0.2
7.0	1.9	0.00	1.9	0.95	0.95	1.55	0.35	0.2

To obtain Different dilutions (1:1, 1:3, and 1:10) and different exogenous H_2O_2 concentrations for centrifuged plasma, we used H_2O_2 working solution of 15 μM. Initially 22.7 μL of 3% H_2O_2 is added to 977 μL of assay buffer to prepare 20 mM of stock solution. Then, 1.5 μL is taken from the prepared stock of 20mM and added to 1998 μL of assay buffer to get the working solution of 15 μM. The flow rates used for undiluted centrifuged plasma, buffer, probe and H_2O_2 stock is as shown in Table S2.

S5: Limit of detection and sensitivity

From the results for the integrated device (see Fig. S4), we observed a distinct FL intensity value at 0.05 µM compared to 0 µM, but the intensity values at 0.025 µM were found to be the same as that at 0 µM. So we can confirm the LOD of the system is 0.05 µM. The sensitivity is calculated as the slope of the intensity vs. concentration graph shown in Fig. 5.

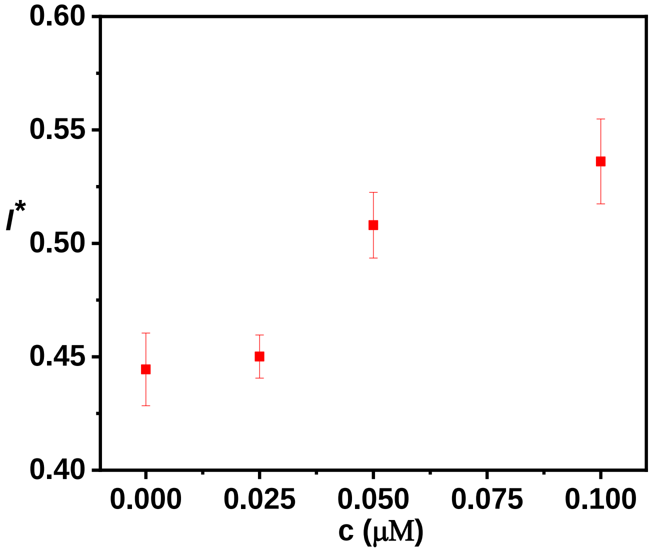

Fig. S4 Variation of FL intensity with H_2O_2 concentration in plasma measured using the integrated system. (Each data point represent the standard deviation (SD) from three different readings)

References

1 S. Karthick and A. K. Sen, Improved Understanding of Acoustophoresis and Development of an Acoustofluidic Device for Blood Plasma Separation, *Phys. Rev. Appl.*, DOI:10.1103/PhysRevApplied.10.034037.

2 I. R. G. Ogilvie, V. J. Sieben, C. F. A. Floquet, R. Zmijan, M. C. Mowlem and H. Morgan, Reduction of surface roughness for optical quality microfluidic devices in PMMA and COC, *J. Micromechanics Microengineering*, , DOI:10.1088/0960-1317/20/6/065016.
